# Treatment patterns and outcomes in older patients with advanced malignant pleural mesothelioma: Analyses of Surveillance, Epidemiology, and End Results‐Medicare data

**DOI:** 10.1002/cnr2.1568

**Published:** 2021-10-26

**Authors:** Mark D. Danese, Melinda Daumont, Esmond Nwokeji, Michelle Gleeson, John R. Penrod, Deborah Lubeck

**Affiliations:** ^1^ Outcomes Insights, Inc. Agoura Hills California USA; ^2^ Bristol Myers Squibb Princeton New Jersey USA

**Keywords:** costs, mesothelioma, overall survival, Surveillance, Epidemiology, and End Results, treatment

## Abstract

**Background:**

Malignant mesothelioma is a rare neoplasm associated with asbestos exposure. Characterizing treatment patterns and outcomes of older patients with advanced malignant pleural mesothelioma (MPM) is important to understand the unmet needs of this population.

**Aim:**

To evaluate the demographic and clinical characteristics, treatment patterns, and outcomes among older patients diagnosed with advanced MPM in the United States between 2007 and 2013.

**Methods:**

This was a retrospective cohort study using Surveillance, Epidemiology, and End Results (SEER) data linked with Medicare claims. We included patients who were age 66 or older at the time of their primary MPM diagnosis between 2007 and 2013 and followed them through 2014. Treated patients who received first‐line chemotherapy with pemetrexed and platinum within 90 days of diagnosis, second‐line, or third‐line therapy were identified for evaluation of outcomes.

**Results:**

There were 666 older patients with advanced MPM, of whom 82% were male, 87% White, 78% stage IV, and 70% had no mobility limitation indicators at diagnosis. There were 262 patients who received first‐line chemotherapy for advanced MPM, most of whom (80%; *n* = 209) received pemetrexed‐platinum. Of these 209 patients, 41% (*n* = 86) initiated second‐line therapy, and 26% (*n* = 22) initiated third‐line therapy. Median overall survival for the cohort of 209 patients was 7.2 months. Patients with epithelioid histology had better median overall survival (12.2 months) compared with other histologies (4.4–5.6 months). Within 90 days of diagnosis of advanced MPM, 78% of patients were hospitalized, 52% visited an emergency department, and 21% had hospice care. The 2‐year cost of care was over $100 000 for all patients with advanced MPM treated with first‐line pemetrexed‐platinum.

**Conclusions:**

Although first‐line systemic anticancer treatment was generally consistent with guidelines (e.g., pemetrexed‐platinum), poor patient outcomes highlight the need for effective treatment options for older patients with advanced MPM.

## 1 INTRODUCTION

Malignant mesothelioma is a rare neoplasm associated with asbestos exposure.^1^ It arises from mesothelial surfaces of the pleural cavity, peritoneal cavity, or pericardium. About 3300 new cases of mesothelioma are diagnosed each year in the United States.^2^ Malignant pleural mesothelioma (MPM) is the most common type and can be difficult to treat because most patients have advanced disease at diagnosis.^2^ MPM presents with gradually worsening, nonspecific pulmonary symptoms, typically in patients older than 60 years of age, decades after exposure to asbestos.^3^ Criteria for staging MPM have been developed, but are regarded as difficult to apply accurately before surgery in clinical practice.^4^, ^5^ The prognosis of patients with advanced MPM is poor, with overall survival (OS) ranging from 9 to 17 months after diagnosis.^6^ Mortality varies by underlying histology, with the epithelioid subtype associated with the longest median OS (11.1 months) and fibrous subtypes, the shortest (3.6 months).^7^

Historically, the main treatment options for MPM included surgery, chemotherapy, and radiation therapy.^5^ Although surgery is associated with improved survival,^6^ it is generally not an option for patients with advanced MPM.^8^ The National Comprehensive Cancer Network (NCCN) Clinical Practice Guidelines in Oncology (NCCN Guidelines®) and the American Society for Clinical Oncology (ASCO) Clinical Practice Guideline recommend chemotherapy as part of a multimodal regimen for medically operable MPM, or as a single modality treatment in patients with advanced or unresectable MPM.^5^, ^8^ Despite the large proportion of patients requiring chemotherapy, there are limited systemic therapy options available to patients. The combination of pemetrexed and cisplatin is a recommended first‐line treatment for patients with unresectable MPM according to the NCCN Guidelines® and the ASCO guideline, and was the only US Food and Drug Administration (FDA)‐approved regimen in this setting until recently.^5^, ^8^ In October 2020, nivolumab in combination with ipilimumab was approved as first‐line treatment for adult patients with unresectable MPM, based on the findings of CheckMate 743, a randomized, open‐label Phase 3 trial that demonstrated a statistically significant OS improvement with nivolumab plus ipilimumab versus chemotherapy in this treatment setting.^9^, ^10^ Accordingly, nivolumab plus ipilimumab is recommended (category 1) in the current NCCN Guidelines as a preferred first‐line treatment option for patients with unresectable biphasic or sarcomatoid MPM; nivolumab plus ipilimumab is also an option (category 1) for patients with epithelioid histology.^5^, ^8^, ^9^ Pemetrexed and cisplatin, with or without bevacizumab, is another preferred first‐line treatment option for patients with MPM (category 1).^5^, ^8^ Other recommendations by the NCCN Guidelines for first‐line treatment include addition of bevacizumab to pemetrexed plus carboplatin, with or without maintenance bevacizumab, gemcitabine plus cisplatin, or monotherapy with pemetrexed or vinorelbine.^8^ Currently, there are no FDA‐approved treatments for second‐ or later line of therapy.

Characterizing treatment patterns and outcomes of older patients with advanced MPM is important to understand the unmet needs of this population. In this retrospective cohort study, we evaluated the demographic and clinical characteristics, treatment patterns, and outcomes among older patients diagnosed with advanced MPM in the United States between 2007 and 2013.

## 2 METHODS

### 2.1 Study design and data source

This retrospective, observational cohort trial used data from the National Cancer Institute (NCI) Surveillance, Epidemiology, and End Results (SEER) cancer registry linked with Medicare enrollment and claims data. The objectives of this study were to: (1) describe demographic and clinical characteristics of older patients with advanced MPM in the SEER‐Medicare linked database, overall and by line of therapy, with a focus on patients who received first‐line pemetrexed‐platinum; (2) describe systemic treatment patterns in patients receiving second‐line and third‐line therapy following first‐line treatment with pemetrexed‐platinum; (3) estimate OS in these cohorts. An additional, exploratory objective was to describe health care resource utilization and direct costs of care across these cohorts.

The SEER program currently covers ~34% of the US population geographically.^11^ It collects data on incidence of cancer cases within the SEER geographic areas, including patient demographic information, tumor characteristics (e.g., stage, grade, and histology), surgery as part of the initial course of cancer treatment, and mortality. According to SEER, the SEER catchment areas are representative of the demographics of the U.S. population including 31.9% of whites, 30.0% of blacks, 44.0% of Hispanics, 49.3% of American Indian/Alaska Natives, 57.5% of Asians, and 68.5% of Native Hawaiian/Pacific Islanders.^12^ Medicare is the primary payer for virtually all adults age 65 and over in the United States.^13^ The linkage of SEER and Medicare data allows for detailed follow‐up of patients diagnosed with cancer. The SEER‐Medicare linked data include Medicare claims for covered Part A and Part B health care services, including hospital, physician, outpatient, home health, durable medical equipment, and hospice bills. In addition, Medicare provides demographic and mortality data.

### 2.2 Inclusion and exclusion criteria

Adult Medicare patients were identified as having a diagnosis of primary MPM based on *International Classification of Diseases for Oncology* topography code C38.4 between January 1, 2007 and December 31, 2013 (Figure S1). MPM was considered advanced if it was classified as T3, T4, N3, or M1 using the 6th edition of the American Joint Committee on Cancer staging manual.^14^ Furthermore, the cancer had to be the patient's first, primary cancer, and it had to be microscopically confirmed. Patients must have been enrolled in Medicare Part A and Part B for at least 12 months before diagnosis; as a result, because patients enroll in Medicare at age 65, patients must have been aged ≥66 at the time of diagnosis to ensure a sufficient “look‐back” period prior to diagnosis. Patients were excluded for the following reasons: diagnosis by death certificate or autopsy, death in the month of diagnosis, or receipt of systemic therapy before the month of SEER diagnosis.

### 2.3 Study time period

The index date was defined as the first day of the month of diagnosis because a specific day is not provided by SEER. The observation period comprised a baseline period spanning ≥12 months before and including the index date (and no earlier than January 1, 2006), and a follow‐up period beginning immediately after the index date and continuing until death, second primary cancer, enrollment in a Medicare health maintenance organization, or the end of available records (December 31, 2014), whichever came first (Figure S1). Baseline clinical and demographic characteristics were assessed during the baseline period. Outcomes, including patterns of care, were assessed during the follow‐up period.

### 2.4 Study variable definitions

Patient demographic and clinical characteristics, including age, sex, race, indicators related to socioeconomic status, and tumor characteristics, were derived from the SEER and Medicare databases. Histology was categorized as epithelioid, non‐epithelioid (sarcomatoid or biphasic), and mesothelioma not otherwise specified (NOS). Because performance status is not available in SEER, we used a proxy based on claims‐based indicators of mobility limitations, including the use of oxygen and related respiratory therapy supplies, wheelchair and supplies, home health agency use, and skilled nursing facility use. The presence of at least one of these claims‐based indicators of mobility limitations has been identified as an important predictor for outcomes associated with cancer treatment.^15^

Outpatient chemotherapy was defined using Healthcare Common Procedure Coding System codes for infused chemotherapy, as well as National Drug Codes for oral therapies with intravenous equivalents. Systemic therapies for MPM were defined as any agents used by at least three patients to exclude therapies likely to be unrelated to the treatment of pleural mesothelioma.

For each patient, the first line of chemotherapy was defined as any unique agent(s) used in the outpatient setting within 8 days of the first documented systemic agent administration. Subsequent lines were defined as either (1) initiation of a different systemic therapy within 60 days of the complete cessation of all agents from the previous line; or (2) a gap of at least 60 days after receiving first‐line therapy and a subsequent initiation of any systemic therapy, including the previous therapy. Switching from one platinum agent to another was considered to be a continuation of the same line of therapy. Patients untreated with systemic therapy in the outpatient setting (hereafter referred to as “untreated patients”) were defined as those with no evidence of systemic outpatient treatment from diagnosis through day 90 of follow‐up. Patients who received inpatient therapy only, or those who initiated therapy after day 90, were included in this group.

Total healthcare reimbursement and resource utilization were estimated starting from diagnosis for all lines of therapy. For these analyses, costs were defined as Medicare‐reimbursed amounts for all services and were adjusted to 2018 dollars using the US Bureau of Labor Statistics Consumer Price Index. Reimbursed amounts were partitioned into monthly intervals to facilitate accounting for censoring using monthly inverse probability of censoring weights.

### 2.5 Study cohorts

Results were evaluated for the overall cohort of patients with advanced MPM, as well as for the following subgroups based on line of therapy: first‐line systemic therapy with pemetrexed‐platinum, second‐line systemic therapy (a subgroup of first‐line pemetrexed‐platinum), and third‐line systemic therapy (a subgroup of second‐line systemic therapy). Selected results for all first‐line patients, including those who did not receive pemetrexed‐platinum, are provided in Supporting Information.

### 2.6 Statistical analyses

Patient counts less than 11 are not reported to ensure patient privacy, as required by the data use agreement with National Cancer Institute. Categorical variables were summarized by frequencies and proportions, and continuous variables were summarized by means and SDs, or medians and interquartile ranges (IQR) as appropriate. OS over time was calculated using unadjusted Kaplan–Meier estimator. Cox proportional hazards regression was used to identify factors associated with mortality for each cohort.

Cumulative total Medicare reimbursements were calculated using inverse probability of censoring weights.^16^ This process accounts for censored individuals by increasing the weights of uncensored individuals, similar to the Kaplan–Meier estimator.^17^, ^18^ Rates of all‐cause hospitalization, emergency department visits, and hospice utilization were estimated as the proportion of the cohort who utilized each feature within 90 days and within 1 year of diagnosis. All analyses were conducted using R (version 3.4.4).

The study qualified for exemption from federal regulations governing the protection of human subjects based on review by the Quorum Institutional Review Board, Seattle, WA in 2016. Individual patient consent was not required because the data are de‐identified.

## 3 RESULTS

### 3.1 Cohort creation

**FIGURE 1:**
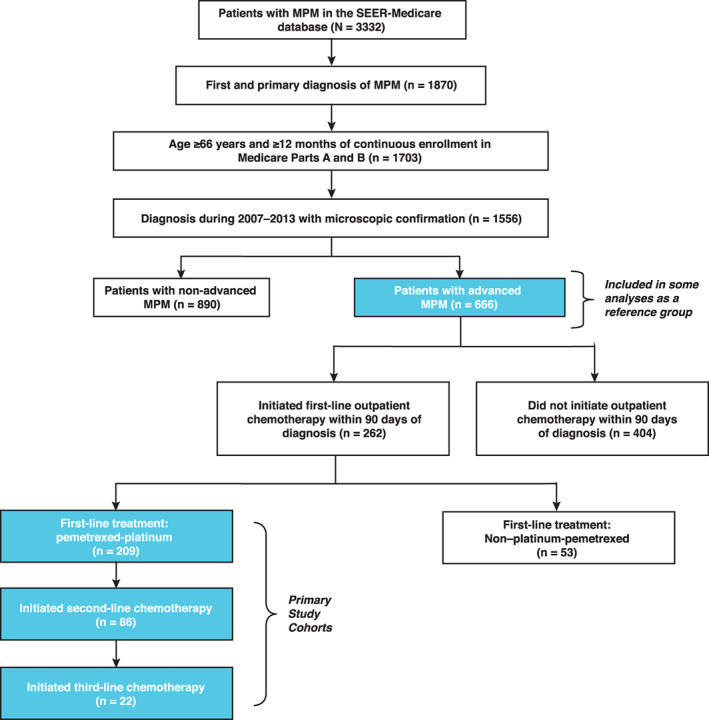
Cohort selection. Patients included in the Surveillance, Epidemiology, and End Results (SEER)‐Medicare linked databases meeting the criteria for inclusion, and treatment patterns among patients included in the study. MPM, malignant pleural mesothelioma

Among 3332 patients with MPM, 1556 (46.7%) met inclusion criteria for age, health plan enrollment, stage, and microscopic confirmation of diagnosis for this study (Figure 1). Of these, 666 (42.8%) met criteria for advanced disease. Of patients with advanced MPM, 262 (39.3%) patients received systemic therapy in an outpatient setting within 90 days of diagnosis, of whom 209 (80%) received pemetrexed‐platinum as first‐line therapy (first‐line pemetrexed‐platinum cohort). Of these, 86 (41%) received subsequent second‐line systemic therapy (second‐line cohort), and of these, 22 (26%) received third‐line therapy (third‐line cohort).

### 3.2 Cohort characteristics

In the overall advanced MPM, first‐line pemetrexed‐platinum, and second‐line cohorts, respectively, mean ages were 77.4, 75.0, and 74.9 years; the proportions of patients age ≥80 years were 37%, 17%, and 16%; the proportions of patients with ≥1 indicator of mobility limitation were 30%, 24%, and 21%; and the proportions with an NCI comorbidity index of ≥2 were 29%, 22%, and 15% (Table 1). Epithelioid histology was present in 37% of the advanced MPM cohort, 44% of the first‐line cohort, and 49% of the second‐line cohort. Non‐epithelioid histology comprised ~23%–24% of all three cohorts. Patients with advanced MPM who received more lines of therapy tended to be healthier at baseline based on age, mobility limitations, and comorbidity burden. Baseline demographics and clinical characteristics for patients with MPM excluded from the primary analysis are reported in Table S1 (all patients with MPM, including subgroups with or without advanced MPM) and Table S2 (patients with advanced MPM, including subgroups who received any first‐line systemic therapy or did not receive therapy).

**TABLE 1:** Baseline demographic and clinical characteristics

Characteristic *n* (%)	Advanced MPM cohort (*n* = 666)	First‐line pemetrexed‐platinum cohort (*n* = 209)	Second‐line cohort^a^ (*n* = 86)
Age			
Mean (SD)	77.4 (6.8)	75.0 (5.4)	74.9 (4.8)
66–<70 years	108 (16.2)	45 (21.5)	18 (20.9)
70–<75 years	153 (23.0)	54 (25.8)	22 (25.6)
75–<80 years	161 (24.2)	74 (35.4)	32 (37.2)
≥80 years	244 (36.6)	36 (17.2)	14 (16.3)
Sex			
Male	546 (82.0)	184 (88.0)	75 (87.2)
Female	120 (18.0)	25 (12.0)	11 (12.8)
Race/ethnicity			
White	581 (87.2)	186 (89.0)	>74 (>86.0)
Black	21 (3.2)	NR	NR
Hispanic	42 (6.3)	11 (5.3)	NR
Other	22 (3.3)	NR	NR
Percent in census tract living in poverty			
0–<5%	194 (29.1)	75 (35.9)	36 (41.9)
5–<10%	180 (27.0)	48 (23.0)	16 (18.6)
10–<20%	184 (27.6)	48 (23.0)	19 (22.1)
≥20%	100 (15.0)	33 (15.8)	13 (15.1)
Geographic area			
Large metropolitan	394 (59.2)	128 (61.2)	51 (59.3)
Metropolitan	183 (27.5)	57 (27.3)	NR
Urban/rural	78 (11.7)	NR	NR
Rural	11 (1.7)	NR	NR
AJCC stage			
III	141 (21.2)	48 (23.0)	19 (22.1)
IV	516 (77.5)	158 (75.6)	67 (77.9)
Histology			
Epithelioid	248 (37.2)	91 (43.5)	42 (48.9)
Non‐epithelioid	159 (23.9)	50 (23.9)	20 (23.2)
NOS	259 (38.9)	68 (32.5)	24 (27.9)
Prior surgery			
No	516 (77.5)	161 (77.0)	64 (74.4)
Yes	149 (22.4)	47 (22.5)	22 (25.6)
Prior radiation			
No	531 (79.7)	158 (75.6)	60 (69.8)
Yes	135 (20.3)	51 (24.4)	26 (30.2)
Indicators of mobility limitations			
0	467 (70.1)	158 (75.6)	68 (79.1)
≥1	199 (29.9)	51 (24.4)	18 (20.9)
NCI comorbidity index			
0	189 (28.4)	101 (48.3)	47 (54.7)
1	197 (29.6)	62 (29.7)	26 (30.2)
≥2	194 (29.1)	46 (22.0)	13 (15.1)
Reason for end of observation			
Death	599 (89.9)	186 (89.0)	>74 (>86.0)
Change in Medicare coverage	33 (5.0)	NR	NR
End of available records	23 (3.5)	NR	NR
Subsequent cancer	11 (1.7)	NR	NR

*Note*: Patients receiving third‐line therapy could not be included due to small sample sizes. Patient counts <11 were not reportable to ensure patient privacy according to the data use agreement for SEER‐Medicare data; totals may not add to 100% due to the omission of these data.

Abbreviations: AJCC, American Joint Committee on Cancer; MPM, malignant pleural mesothelioma; NCI, National Cancer Institute; NOS, not otherwise specified; NR, not reportable; SEER, Surveillance, Epidemiology, and End Results.

^a^ Subgroup of the first‐line pemetrexed‐platinum cohort who received second‐line therapy.

In the first‐line pemetrexed‐platinum cohort, approximately half (47%) of the patients received carboplatin and the remainder received cisplatin as platinum chemotherapy (Table 2). In the second‐line cohort, the most common second‐line regimens were gemcitabine monotherapy (30%) and pemetrexed‐platinum (16%). Treatment regimens among patients with advanced MPM who received any first‐line systemic therapy, including those not treated with pemetrexed‐platinum, are reported in Tables S3 and S4.

**TABLE 2:** Treatment regimens in the first‐line pemetrexed‐platinum and second‐line advanced MPM cohorts

Regimen	*n* (%)
First‐line pemetrexed‐platinum cohort	209 (100)
Pemetrexed‐cisplatin	111 (53.1)
Pemetrexed‐carboplatin	98 (46.9)
Second‐line cohort	86 (100)
Gemcitabine	26 (30.2)
Pemetrexed‐platinum	24 (27.9)
Pemetrexed	14 (16.3)
All others	22 (25.6)

*Note*: Patients receiving third‐line therapy could not be included due to small sample sizes. Patient counts <11 were not reportable to ensure patient privacy according to the data use agreement for SEER‐Medicare data; totals may not add to 100% due to the omission of these data.

Abbreviations: MPM, malignant pleural mesothelioma; SEER, Surveillance, Epidemiology, and End Results.

### 3.3 Overall survival

**FIGURE 2:**
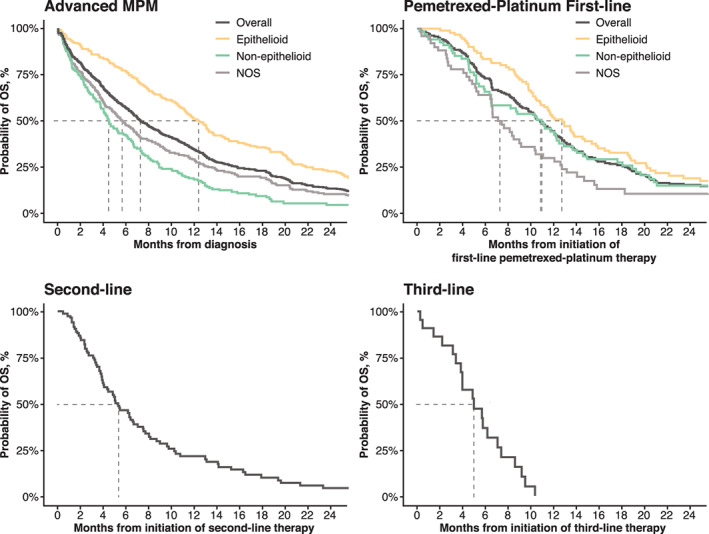
Unadjusted overall survival in study cohorts. (A) All patients with advanced malignant pleural mesothelioma (MPM) (advanced MPM cohort); (B) patients with advanced MPM who received first‐line pemetrexed‐platinum (first‐line pemetrexed‐platinum cohort); (C) subgroup of the first‐line pemetrexed‐platinum cohort who received second‐line therapy (second‐line cohort); and (D) subgroup of the second‐line cohort who received third‐line therapy (third‐line cohort). NOS; not otherwise specified

Approximately 90% of patients with advanced MPM died during follow‐up. Median OS from time of diagnosis for the advanced MPM cohort was 7.2 months (95% CI: 6.6–8.2; Figure 2; Table S5). Median OS was 12.2 months (95% CI: 11.0–13.5) for epithelioid histology, 4.4 months (95% CI: 3.8–6.0) for non‐epithelioid histology, and 5.6 months (95% CI: 4.7–6.8 months) for NOS histology (Figure 2). For the first‐line pemetrexed‐platinum cohort, median OS from the initiation of first‐line therapy was 10.7 months (95% CI: 9.6–12.0). In this cohort, median OS was 13.0 months (95% CI: 11.6–15.7) for epithelioid histology, 7.7 months (95% CI: 6.5–11.3) for non‐epithelioid histology, and 11.3 months (95% CI: 7.8–14.4 months) for NOS histology. Median OS was 5.3 months (95% CI: 4.0–7.0) for the second‐line cohort from the initiation of second‐line therapy and 4.9 months (95% CI: 3.8–7.3) for the third‐line cohort from initiation of third‐line therapy (Figure 2).

For the overall advanced MPM cohort, demographic factors that were significantly associated with worse OS outcomes were (Table S6): older age (hazard ratio [HR] 1.03 per year, 95% CI: 1.02–1.04), mobility limitations (HR 1.33 for any vs. none, 95% CI: 1.10–1.61), higher comorbidity burden (HR 1.23 for 1 vs. 0, 95% CI: 1.00–1.50 and HR 1.65 for 2+ vs. 0, 95% CI: 1.34–2.02), Stage IV diagnosis (HR 1.35 vs. earlier stage, 95% CI: 1.09–1.66) and histology (HR 2.18 for non‐epithelioid vs. non‐epithelioid, 95% CI: 1.74–2.70 and HR 1.41 for NOS vs. epithelioid, 95% CI: 1.16–1.71). Female sex (vs. male) was associated with a lower risk of death (HR 0.79, 95% CI: 0.63–0.98). Factors associated with OS risk for first‐line and second‐line patients are also reported in Table S6.

### 3.4 Treatment duration and resource utilization

Median duration of systemic therapy was 84 days (IQR, 42–136) for the first‐line cohort, 55 days (IQR 33–102) for the second‐line cohort, and 52 days (IQR 24–77) for the third‐line cohort (Table 3). Within 90 days of diagnosis, 52% of patients in the advanced MPM cohort visited an emergency department, 78% were hospitalized, and 21% received hospice care (Table 4). Patients who received second‐ or third‐line therapy had higher cumulative Medicare reimbursed costs than the other cohorts, and all three systemic therapy cohorts incurred 24‐month Medicare reimbursed costs exceeding $100 000 in 2018 dollars (Figure S2).

**TABLE 3:** Treatment patterns in advanced MPM patient cohorts

	*n*	Time from diagnosis to treatment, days Median (IQR)	Duration of treatment, days Median (IQR)	Time between lines of therapy, days Median (IQR)
First‐line pemetrexed‐platinum cohort	209	50 (38–66)	84 (42–136)	NA
Second‐line cohort	86	296 (203–420)	55 (33–102)	105 (46–188)
Pemetrexed‐platinum	24	323 (202–486)	61 (42–102)	178 (112–311)
Gemcitabine	26	280 (218–357)	48 (34–92)	67 (23–112)
Pemetrexed	14	430 (310–512)	56 (12–108)	166 (124–328)
Other	22	233 (142–388)	56 (30–106)	56 (30–105)
Third‐line cohort	22	512 (292–732)	52 (24–77)	56 (28–180)

*Note*: Patient counts <11 were not reportable to ensure patient privacy according to the data use agreement for SEER‐Medicare data; totals may not add to 100% due to the omission of these data.

Abbreviations: IQR, interquartile range; MPM, malignant pleural mesothelioma; NA, not applicable; NR, not reportable; SEER, Surveillance, Epidemiology, and End Results.

**TABLE 4:** Healthcare resource utilization in advanced MPM cohorts

*n* (%)	Advanced MPM cohort (*n* = 666)	First‐line pemetrexed‐platinum cohort (*n* = 209)	Second‐line cohort (*n* = 86)
Hospitalization within 90 days of diagnosis	518 (77.8)	163 (78.0)	65 (75.6)
Hospitalization within 1 year of diagnosis	569 (85.4)	187 (89.5)	75 (87.2)
ED within 90 days of diagnosis	344 (51.7)	98 (46.9)	39 (45.4)
ED within 1 year of diagnosis	469 (70.4)	154 (73.7)	61 (70.9)
Hospice within 90 days of diagnosis	141 (21.2)	NR	NR
Hospice within 1 year of diagnosis	325 (48.8)	85 (40.7)	23 (26.7)

*Note*: Patients receiving third‐line therapy could not be included due to small sample sizes. Patient counts <11 were not reportable to ensure patient privacy according to the data use agreement for SEER‐Medicare data; totals may not add to 100% due to the omission of these data.

Abbreviations: ED, emergency department; MPM, malignant pleural mesothelioma; NR, not reportable; SEER, Surveillance, Epidemiology, and End Results.

## 4 DISCUSSION

Although a number of studies have evaluated specific treatment patterns and/or survival in patients with pleural mesothelioma in the United States,^19^, ^20^, ^21^ ours is unique in that it describes treatment by lines of therapy, OS, resource utilization, and costs in older patients with MPM. Patients diagnosed with MPM between 2007 and 2013 had poor OS, with half of patients dying by just over 7 months from diagnosis. Patients with epithelioid histology had better median OS (12.2 months) compared with other histologies (4.4–5.6 months). The majority of patients initiating first‐line therapy received pemetrexed‐platinum, consistent with guideline recommendations in place at the time of data collection. Gemcitabine monotherapy and pemetrexed‐platinum were the most common second‐line regimens. Most patients (78%) were hospitalized, 52% visited an emergency department, and 21% received hospice care. The 2‐year cost of care was over $100 000 for all patients treated with systemic therapy, and was over $75 000 for those who were not. Overall, the prognosis for MPM was poor.

Other studies have used the SEER data (without the Medicare linkage) to evaluate factors associated with OS including histology.^6^, ^7^, ^22^ These also showed consistently poor OS in mesothelioma and that epithelioid histology was associated with the best OS, followed by NOS and then non‐epithelioid histology. Because these were limited to SEER data, they could not evaluate systemic therapy or the effects of risk factors unrelated to cancer or its treatment (e.g., comorbidity, mobility limitations, and rural/urban status).

There is one published study of older patients with malignant mesothelioma using the SEER‐Medicare linked data from 2005 to 2009; however, it was not restricted to advanced MPM and included patients with lung or peritoneal mesothelioma, and it did not identify factors associated with OS.^23^ The study identified a cohort of 1625 patients, similar in size to our cohort, and reported that 55% of patients with MPM did not initiate chemotherapy, similar to our estimate of 60% for advanced MPM. In that study, the most common first‐line treatment was a platinum‐based agent in combination with pemetrexed (67%). The median OS in that study was 8 months, with a 1‐year OS rate of 31%; whereas in our cohort of advanced MPM, median OS for all patients was 7.2 months, with a 34% OS rate at 1 year.^23^ Overall, the results of the previous SEER‐Medicare study were consistent with the findings from our study, suggesting that there was little change in patterns of care or outcomes in patients diagnosed from 2005 through 2013. Given the recent approval of nivolumab in combination with ipilimumab and its inclusion in the NCCN Guidelines,^8^ it will be interesting to determine how treatment patterns and outcomes change over coming years.

It is important to keep in mind that it is difficult to compare the OS results from our study (median OS of 10.7 months in the first‐line pemetrexed‐platinum population that included patients 66 years of age or older regardless of metastases or functional status, 63% of whom had non‐epithelioid or NOS histology) with those from recent randomized controlled trials because of the differences in the inclusion and exclusion criteria. For example, 28% of the trial population in CheckMate 743 were younger than 65 years, the trial excluded patients with brain metastases or with poor functional status, and most patients (75%) had epithelioid histology.^10^ In the chemotherapy arm of CheckMate 743, median OS was 14.1 months in the overall population and 8.8 months in the non‐epithelioid subgroup. We should note that in CheckMate 743, median OS in patients 75 years of age or older was similar between the treatment groups.

This study is strengthened by its broad coverage of US cancer patients; the availability of cancer location, histology, behavior, grade, and stage from SEER; accurate information about date of death; and the availability of comprehensive Medicare claims data that include direct health care expenditures across all settings of care (inpatient, outpatient, pharmacy, and home care). Furthermore, the availability of Medicare claims data allows for the identification of additional clinical and socioeconomic factors associated with OS and for identifying lines of therapy.

As with any study using observational data, there are limitations and other factors to be considered when interpreting these findings. Our focus was on patients with advanced MPM who received platinum‐pemetrexed as first‐line therapy, which was the most commonly used first‐line treatment at the time of the study. Additional information for earlier‐stage patients and for patients who received other first‐line therapy (primarily pemetrexed monotherapy) is provided in Supporting Information. Specific systemic therapy agents provided in the hospital were not identifiable; hence, these patients were not included in our “treated” cohorts. Our cohort was limited to Medicare enrollees age ≥66 years diagnosed through 2013 in SEER catchment areas. These analyses may be less relevant for younger patients, and may not reflect the most recent treatment trends due to the time lag (data through December 31, 2014). Neither SEER nor Medicare data capture information about non‐covered services or reasons for patient treatment choices. Because this study focused on older patients with advanced MPM, the advanced nature of mesothelioma and comorbid conditions may have affected the choice to use systemic therapy and may have been associated with worse OS compared with the overall mesothelioma population. Finally, we excluded patients who did not have staging information available.

In conclusion, in older patients with advanced MPM, low treatment rates and poor OS were observed across lines of therapy. Although most patients received treatment consistent with guidelines (e.g., pemetrexed‐platinum), their poor outcomes highlight the areas where more effective treatment options could benefit older patients with advanced MPM.

## CONFLICT OF INTEREST

Mark D. Danese, Michelle Gleeson, and Deborah Lubeck are employees of Outcomes Insights, Inc, which was funded by Bristol‐Myers Squibb for performing the analysis reported in this manuscript. Melinda Daumond and John R. Penrod are employees of Bristol‐Myers Squibb. Esmond Nwokeji was a BMS contractor at the time this research was conducted.

## AUTHOR CONTRIBUTIONS

**Mark Danese:** Conceptualization (lead); data curation (lead); formal analysis (lead); writing – original draft (lead); writing – review and editing (lead). **Melinda Manley Daumont:** Conceptualization (supporting); writing – review and editing (supporting). **Esmond Nwokeji:** Conceptualization (supporting); writing – review and editing (supporting). **Michelle Gleeson:** Conceptualization (supporting); data curation (supporting); formal analysis (supporting); writing – review and editing (supporting). **John R. Penrod:** Conceptualization (supporting); writing – review and editing (supporting). **Deborah Lubeck:** Conceptualization (lead); data curation (lead); formal analysis (lead); writing – review and editing (lead).

## ETHICS STATEMENT

The study was exempted from institutional review board (IRB) approval because it used de‐identified patient data (Quorum IRB, Protocol Exemption Determination 31309).

## Supporting information


**Appendix S1**: Supporting Information

## Data Availability

The Data Use Agreement for the SEER‐Medicare data do not permit sharing of the data.
