# Supplementary material for: Treatment patterns and outcomes in older patients with advanced malignant pleural mesothelioma: Analyses of Surveillance, Epidemiology, and End Results‐Medicare data
Source: Cancer Rep (Hoboken). 2021 Oct 26;5(9):e1568. doi: 10.1002/cnr2.1568 (PMC9458508; doi:10.1002/cnr2.1568)
Supplement: Supplementary file 1 — Appendix S1: Supporting Information [file CNR2-5-e1568-s001.docx]

**Supplementary Materials**

Treatment Patterns and Outcomes in Advanced Malignant Pleural Mesothelioma: Surveillance, Epidemiology, and End Results (SEER)-Medicare Linked Data

**Supplementary Figure S1** Cohort creation process


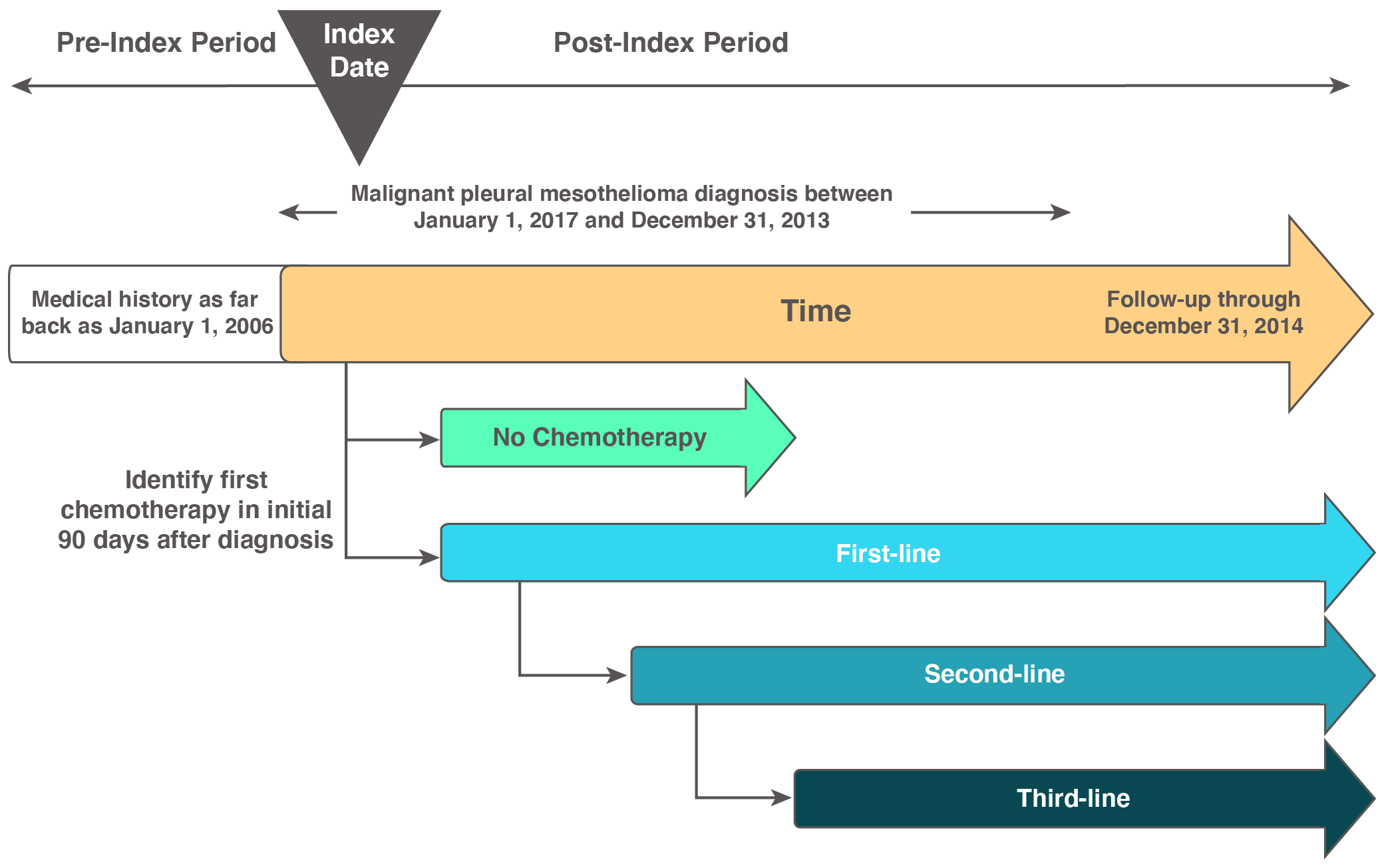


**Supplementary Figure S2** Unadjusted cumulative reimbursement (2018 US$) for the advanced MPM, first-line pemetrexed-platinum, second-line, and third-line cohorts. Note: Cost accumulation begins at diagnosis for all cohorts. MPM, malignant pleural mesothelioma


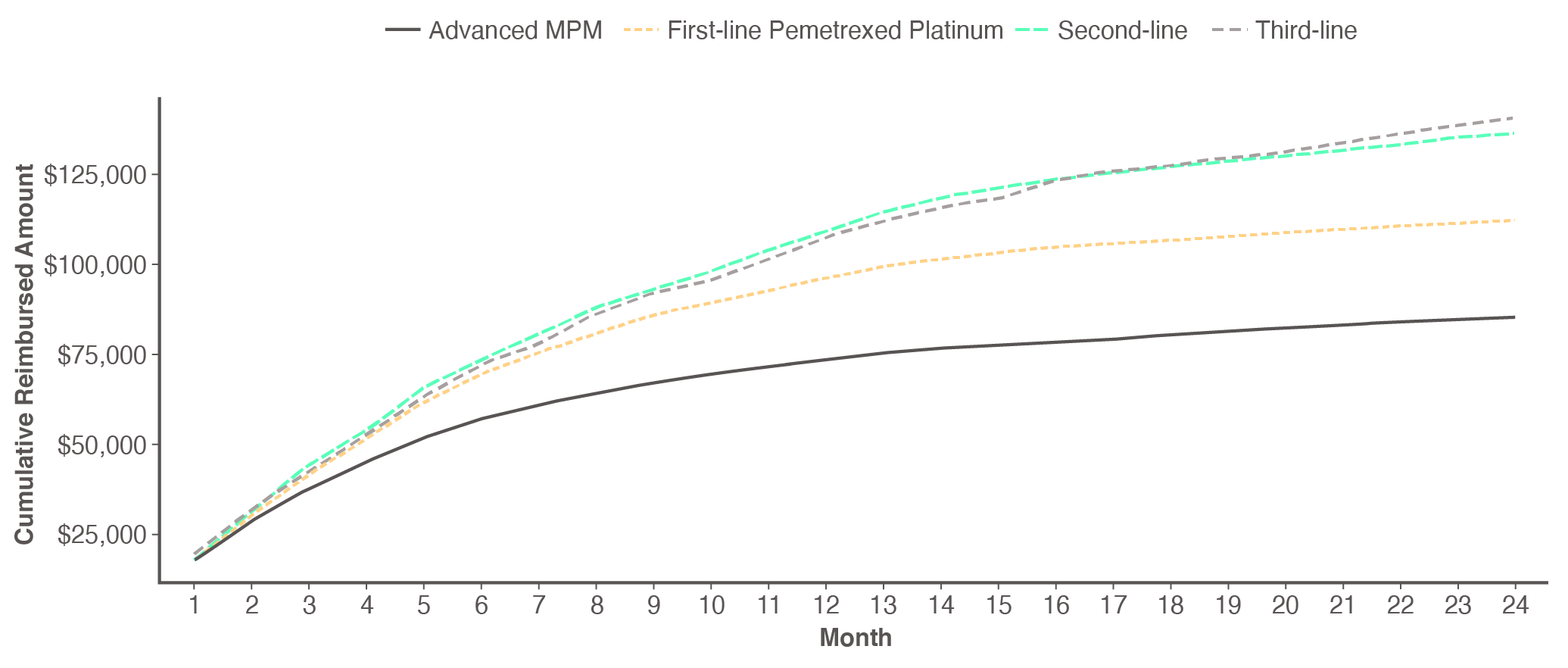


**Supplementary Table S1** Baseline demographics and clinical characteristics of all patients with advanced and non-advanced MPM

| **Characteristic**  **n (%)** | **All MPM (n = 1556)** | **Advanced MPM Cohort (n = 666)** | **Patients With**  **Non‐Advanced MPM  (n = 890)** |
| --- | --- | --- | --- |
| **Age** |  |  |  |
| 66–69 years | 221 (14.2) | 108 (16.2) | 113 (12.7) |
| 70–74 years | 324 (20.8) | 153 (23.0) | 171 (19.2) |
| 75–79 years | 370 (23.8) | 161 (24.2) | 209 (23.5) |
| ≥ 80 years | 641 (41.2) | 244 (36.6) | 397 (44.6) |
| **Sex** |  |  |  |
| Male | 1243 (79.9) | 546 (82.0) | 697 (78.3) |
| Female | 313 (20.1) | 120 (18.0) | 193 (21.7) |
| **Race/ethnicity** |  |  |  |
| White | 1385 (89.0) | 581 (87.2) | 804 (90.3) |
| Black | 49 (3.1) | 21 (3.2) | 28 (3.1) |
| Hispanic | 77 (4.9) | 42 (6.3) | 35 (3.9) |
| Other | 45 (2.9) | 22 (3.3) | 23 (2.6) |
| **Percent in census tract living in poverty** |  |  |  |
| 0–5% | 476 (30.6) | 194 (29.1) | 282 (31.7) |
| 5–10% | 416 (26.7) | 180 (27.0) | 236 (26.5) |
| 10–20% | 423 (27.2) | 184 (27.6) | 239 (26.9) |
| ≥ 20% | 228 (14.7) | 100 (15.0) | 128 (14.4) |
| **Geographic area** |  |  |  |
| Large metropolitan | 892 (57.3) | 394 (59.2) | 498 (56.0) |
| Metropolitan | 444 (28.5) | 183 (27.5) | 261 (29.3) |
| Urban | 187 (12.0) | 78 (11.7) | 109 (12.2) |
| Rural | 33 (2.1) | 11 (1.7) | 22 (2.5) |
| **AJCC Stage** |  |  |  |
| I | 312 (20.1) | 0 | 312 (35.1) |
| II | 201 (12.9) | 0 | 201 (22.6) |
| III | 274 (17.6) | > 139 (> 20.9) | < 135 (> 15.2) |
| IV | 516 (33.2) | 516 (77.5) | 0 |
| Other/unknown | 253 (16.3) | < 11 (< 1.7) | > 242 (> 27.2) |
| **Histology** |  |  |  |
| Epithelioid | 574 (36.9) | 248 (37.2) | 326 (36.6) |
| Non-epithelioid | 317 (20.4) | 159 (23.9) | 158 (17.8) |
| NOS | 665 (42.7) | 259 (38.9) | 406 (45.6) |
| **Prior surgery** |  |  |  |
| No | 1216 (78.1) | 516 (77.5) | 700 (78.7) |
| Yes | 329 (21.1) | 149 (22.4) | 180 (20.2) |
| **Prior radiation** |  |  |  |
| No | 1330 (85.5) | 531 (79.7) | 799 (89.8) |
| Yes | 226 (14.5) | 135 (20.3) | 91 (10.2) |
| **Indicators of mobility limitations** |  |  |  |
| 0 | 1026 (65.9) | 467 (70.1) | 559 (62.8) |
| ≥ 1 | 530 (34.1) | 199 (29.9) | 331 (37.2) |
| **NCI comorbidity index** |  |  |  |
| 0 | 600 (38.6) | 280 (42.0) | 320 (36.0) |
| 1 | 463 (29.8) | 189 (28.4) | 274 (30.8) |
| ≥ 2 | 493 (31.7) | 197 (29.6) | 296 (33.3) |
| **Reason for end of observation** |  |  |  |
| Death | 1390 (89.3) | 599 (89.94) | 791 (88.9) |
| Change in Medicare coverage | 78 (5.0) | 33 (4.95) | 45 (5.1) |
| End of available records | 65 (4.2) | 23 (3.45) | 42 (4.7) |
| Subsequent cancer | 23 (1.5) | 11 (1.7) | 12 (1.3) |

AJCC, American Joint Committee on Cancer; MPM, malignant pleural mesothelioma; NCI, National Cancer Institute; NOS, not otherwise specified; NR, not reportable; SEER, Surveillance, Epidemiology, and End Results

Patient counts < 11 data points were not reportable to ensure patient privacy according to the data use agreement for SEER-Medicare data; totals may not add to 100% due to the omission of these data.

**Supplementary Table S2** Baseline demographics and clinical characteristics of all treated and untreated patients with advanced MPM

| **Characteristic**  **Frequency (%)** | **Advanced MPM cohort (n = 666)** | | **Treated**  **(n = 262)** | **Untreated**  **(n = 404)** |
| --- | --- | --- | --- | --- |
| **Age** |  | |  |  |
| 66–69 years | 108 (16.2) | | 50 (19.1) | 58 (14.4) |
| 70–74 years | 153 (23.0) | | 69 (26.3) | 84 (20.8) |
| 75–79 years | 161 (24.2) | | 87 (33.2) | 74 (18.3) |
| ≥ 80 years | 244 (36.6) | | 56 (21.4) | 188 (46.5) |
| **Sex** |  | |  |  |
| Male | 546 (82.0) | | 223 (85.1) | 323 (80.0) |
| Female | 120 (18.0) | | 39 (14.9) | 81 (20.0) |
| **Race/ethnicity** |  | |  |  |
| White | 581 (87.2) | | 234 (89.3) | 347 (85.9) |
| Black | 21 (3.2) | | NR | NR |
| Hispanic | 42 (6.3) | | 13 (5.0) | 29 (7.2) |
| Other | 22 (3.3) | | NR | NR |
| **Percent in census tract living in poverty** |  | |  |  |
| 0–5% | 194 (29.1) | | 91 (34.7) | 103 (25.5) |
| 5–10% | 180 (27.0) | | 62 (23.7) | 118 (29.2) |
| 10–20% | 184 (27.6) | | 63 (24.0) | 121 (30.0) |
| ≥ 20% | 100 (15.0) | | 41 (15.6) | 59 (14.6) |
| **Geographic area** |  | |  |  |
| Large metropolitan | 394 (59.2) | | 160 (61.1) | 234 (57.9) |
| Metropolitan | 183 (27.5) | | 70 (26.7) | 113 (28.0) |
| Urban/rural | 78 (11.7) | | NR | NR |
| Rural | 11 (1.7) | | NR | NR |
| **AJCC stage** |  | |  |  |
| III | 141 (21.2) | | 52 (19.8) | 89 (22.0) |
| IV | 516 (77.5) | | 206 (78.6) | 310 (76.7) |
| **Histology** |  |  | |  |
| Epithelioid | 248 (37.2) | 110 (42.0) | | 138 (34.2) |
| Non-epithelioid | 159 (23.9) | 62 (23.7) | | 97 (24.0) |
| NOS | 259 (38.9) | 90 (34.4) | | 169 (41.8) |
| **Prior surgery** |  | |  |  |
| No | 516 (77.5) | | 209 (79.8) | 307 (76.0) |
| Yes | 149 (22.4) | | 52 (19.8) | 97 (24.0) |
| **Prior radiation** |  | |  |  |
| No | 531 (79.7) | | 198 (75.6) | 333 (82.4) |
| Yes | 135 (20.3) | | 64 (24.4) | 71 (17.6) |
| **Indicators of mobility limitations** |  | |  |  |
| 0 | 467 (70.1) | | 198 (75.6) | 269 (66.6) |
| ≥ 1 | 199 (29.9) | | 64 (24.4) | 135 (33.4) |
| **NCI comorbidity index** |  | |  |  |
| 0 | 189 (28.4) | | 74 (28.2) | 115 (28.5) |
| 1 | 197 (29.6) | | 59 (22.5) | 138 (34.2) |
| ≥ 2 | 194 (29.1) | | 91 (34.7) | 103 (25.5) |
| **Reason for end of observation** |  | |  |  |
| Death | 599 (89.9) | | 230 (87.8) | 369 (91.3) |
| Change in Medicare coverage | 33 (5.0) | | 14 (5.3) | 19 (4.7) |
| End of available records | 23 (3.5) | | NR | NR |
| Subsequent cancer | 11 (1.7) | | NR | NR |

AJCC, American Joint Committee on Cancer; MPM, malignant pleural mesothelioma; NCI, National Cancer Institute; NOS, not otherwise specified; NR, not reportable; SEER, Surveillance, Epidemiology, and End Results

Patients receiving outpatient systemic therapy within 90 days of diagnosis were classified as “treated”. All other patients were classified as “untreated” for this table, but may have received other therapy or received systemic therapy after day 90. Patient counts < 11 were not reportable to ensure patient privacy according to the data use agreement for SEER-Medicare data; totals may not add to 100% due to the omission of these data.

**Supplementary Table S3** Treatment regimens in all treated patients with advanced MPM

| **Regimen** | **N (%)** |
| --- | --- |
| **Any first-line treatment^†^** | 262 (100) |
| Cisplatin-pemetrexed | 111 (42.4) |
| Carboplatin-pemetrexed | 98 (37.4) |
| Pemetrexed | 35 (13.4) |
| Other | 18 (6.8) |
| **Second-line treatment^‡^** | 106 (100) |
| Gemcitabine | 30 (28.3) |
| Carboplatin-pemetrexed | 20 (18.9) |
| Pemetrexed | 19 (17.9) |
| Cisplatin-pemetrexed | 11 (10.4) |
| Other | 26 (24.5) |
| **Third-line treatment^§^** | 29 (100) |

MPM, malignant pleural mesothelioma; SEER, Surveillance, Epidemiology, and End Results

^†^Includes patients who received any systemic therapy as first-line treatment (not limited to patients treated with first-line pemetrexed-platinum). ^‡^Subgroup of patients who received second-line therapy after any first-line therapy. ^§^Subgroup of patients who received third-line therapy after any first- and second-line therapy.

Patient counts < 11 were not reportable to ensure patient privacy according to the data use agreement for SEER-Medicare data; totals may not add to 100% due to the omission of these data.

**Supplementary Table S4** Second-line chemotherapy regimens in all treated patients with advanced MPM^†^

| **First-line regimen** | **n for first-line**  **(N = 262)** | **Second-line regimen** | **n for second-line** |
| --- | --- | --- | --- |
| Pemetrexed-carboplatin | 98 | None | 57 |
|  |  | Gemcitabine | 17 |
|  |  | All other | 24 |
| Pemetrexed-cisplatin | 111 | None | 66 |
|  |  | Pemetrexed or gemcitabine | 19 |
|  |  | Pemetrexed-platinum | 16 |
| Pemetrexed | 35 | None | 22 |
|  |  | All other | 13 |
| All other | 18 | None | NR |
|  |  | All other | NR |

MPM, malignant pleural mesothelioma; NR, not reportable; SEER, Surveillance, Epidemiology, and End Results

^†^Includes patients who received second-line therapy after any systemic therapy as first-line treatment (**not** limited to patients treated with first-line pemetrexed platinum).

Patient counts < 11 were not reportable to ensure patient privacy according to the data use agreement for SEER-Medicare data; totals may not add to 100% due to the omission of these data.

**Supplementary Table S5** Summary statistics for unadjusted overall survival by cohort

| **Cohort** | **N** | **Median OS, months (95% CI)** | **2-Year restricted mean OS, months^†^ (95% CI)** | **1-Year OS rate^‡^**  **(95% CI)** | **2-Year OS rate^‡^ (95% CI)** |
| --- | --- | --- | --- | --- | --- |
| Advanced MPM | 666 | 7.2 (6.6–8.2) | 9.8 (6.6–8.2) | 0.34 (0.30–0.38) | 0.14 (0.11–0.17) |
| First-line pemetrexed-platinum cohort | 209 | 10.7 (9.6–12.0) | 11.8 (9.6–12.0) | 0.43 (0.36–0.50) | 0.15 (0.11–0.22) |
| Second-line cohort | 86 | 5.3 (4.0–7.0) | 7.6 (4.0–7.0) | 0.22 (0.14–0.33) | 0.04 (0.01–0.13) |
| Third-line cohort | 22 | 4.9 (3.8–7.3) | 5.2 (3.8–7.3) | -- | -- |

CI, confidence interval; MPM, malignant pleural mesothelioma; OS, overall survival.

^†^Restricted mean OS is the mean number of months of survival during the first 2 years after the index date. OS is measured from the index date for each cohort (diagnosis or the initiation of the line of therapy, as appropriate). ^‡^Proportion surviving, range 0 to 1.

**Supplementary Table S6** Adjusted overall survival by cohort

| **Variable** | **Level** | **Advanced MPM** | | **First-line pemetrexed-platinum cohort^†^** | | **Second-line cohort^†^** | |
| --- | --- | --- | --- | --- | --- | --- | --- |
|  |  | **Hazard ratio (95% CI)** | ***P*-value** | **Hazard ratio (95% CI)** | ***P*-value** | **Hazard ratio**  **(95% CI)** | ***P*-value** |
| Age | Per 1 year | 1.03  (1.02–1.04) | < 0.01 | 1.00  (0.97–1.03) | 0.91 | 0.96  (0.91–1.02) | 0.17 |
| Sex | Female vs. male | 0.79  (0.63–0.98) | 0.04 | 0.65  (0.40–1.07) | 0.09 | 0.77  (0.32–1.86) | 0.56 |
| Race | Black vs. White | 1.07  (0.65–1.75) | 0.80 | 0.96  (0.33–2.85) | 0.94 | 1.52  (0.13–17.49) | 0.74 |
|  | Other vs. White | 1.18  (0.89–1.56) | 0.25 | 1.48  (0.87–2.49) | 0.15 | 1.31  (0.49–3.48) | 0.59 |
| Stage | IV vs. all else | 1.35  (1.09–1.66) | < 0.01 | 1.51  (1.02–2.25) | 0.04 | 2.07  (1.01–4.25) | 0.05 |
| Urban/rural status | Urban/metro vs. large metropolitan | 1.04  (0.87–1.24) | 0.68 | 0.81  (0.58–1.14) | 0.23 | 0.88  (0.49–1.57) | 0.66 |
|  | Rural vs. large metropolitan | 1.85  (0.95–3.60) | 0.07 | 0.89  (0.20–4.04) | 0.88 | 0.34  (0.03–3.70) | 0.38 |
| Mobility limitations | At least 1 vs. none | 1.33  (1.10–1.61) | < 0.01 | 1.39  (0.95–2.03) | 0.09 | 0.92  (0.42–2.03) | 0.84 |
| NCI Comorbidity Index | Score 1 vs. 0 | 1.23  (1.00–1.50) | 0.05 | 1.18  (0.82–1.71) | 0.37 | 0.86  (0.42–1.75) | 0.68 |
|  | Score 2+ vs. 0 | 1.65  (1.34–2.02) | < 0.01 | 1.10  (0.72–1.68) | 0.65 | 1.32  (0.54–3.25) | 0.54 |
| Poverty | Census poverty level  5–< 10% vs. 0–< 5% | 1.02  (0.82–1.27) | 0.85 | 1.07  (0.72–1.61) | 0.73 | 0.85  (0.37–1.97) | 0.71 |
|  | Census poverty level  10–<20% vs. 0–5% | 0.99  (0.80–1.24) | 0.94 | 0.92  (0.61–1.40) | 0.71 | 0.79  (0.38–1.64) | 0.53 |
|  | Census poverty level ≥ 20% & missing vs. 0–< 5% | 1.14  (0.86–1.50) | 0.37 | 1.12  (0.69–1.81) | 0.65 | 1.35  (0.56–3.25) | 0.50 |
| Histology | Non-epithelioid vs. epithelioid | 2.18  (1.75–2.70) | < 0.01 | 1.67  (1.14–2.44) | 0.01 | 1.03  (0.52–2.03) | 0.94 |
|  | NOS vs. epithelioid | 1.41 (1.16–1.71) | < 0.01 | 1.01  (0.69–1.46) | 0.96 | 0.76  (0.39–1.45) | 0.40 |

CI, confidence interval; MPM, malignant pleural mesothelioma; NCI, National Cancer Institute; NOS, not otherwise specified.

^†^Results from Cox proportional hazards models starting at diagnosis, initiation of first-line therapy, and initiation of second-line therapy, as appropriate.
